# Poly[bis­[8-ethyl-5-oxo-2-(piperazin-1-yl)-5,8-dihydro­pyrido[2,3-*d*]pyrimidine-6-carboxyl­ato]cadmium]

**DOI:** 10.1107/S1600536810043291

**Published:** 2010-10-31

**Authors:** Jun Gao, Jing Gao, Bo Pei, Jing Huang

**Affiliations:** a School of Physical Education and Sports Science, Harbin Normal University, Harbin 150025, People’s Republic of China; b Department of Pharmacy, Mudanjiang Medical University, Mudanjiang 157011, People’s Republic of China; c School of Chemistry and Chemical Engineering, Harbin Normal University, Harbin 150025, People’s Republic of China; d School of Pharmaceutical Science, Harbin Medical University, Harbin 150086, People’s Republic of China

## Abstract

The title layered coordination polymer, [Cd(C_14_H_16_N_5_O_3_)_2_]_*n*_ or [Cd(ppa)_2_]_*n*_, where ppa is 8-ethyl-5-oxo-2-(piperazin-1-yl)-5,8-dihydro­pyrido[2,3-*d*]pyrimidine-6-carboxyl­ate, was syn­thesized under hydro­thermal conditions. The Cd^II^ atom (site symmetry 2) exhibits a distorted *cis*-CdN_2_O_4_ octa­hedral geometry defined by two *N*-monodentate and two *O*,*O*′-bidentate ppa monoanions. The extended two-dimensional structure resulting from the bridging ppa species is a grid lying parallel to (001). An N—H⋯O hydrogen bond helps to establish the crystal packing.

## Related literature

For the manganese(II), zinc(II), cobalt(II) and nickel(II) complexes of the ppa anion, see: Huang *et al.* (2008 ▶); Xu *et al.* (2009 ▶); Qi *et al.* (2009 ▶); An & Zhu (2010 ▶). For background on the medicinal uses of pipemidic acid, see: Mizuki *et al.* (1996 ▶).
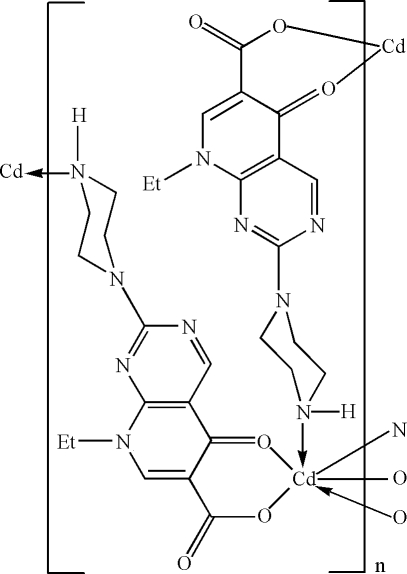


## Experimental

### 

#### Crystal data

[Cd(C_14_H_16_N_5_O_3_)_2_]
*M* *_r_* = 717.04
Monoclinic, 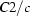

*a* = 23.565 (3) Å
*b* = 7.4989 (10) Å
*c* = 18.719 (3) Å
β = 124.133 (2)°
*V* = 2738.0 (6) Å^3^
*Z* = 4
Mo *K*α radiation
μ = 0.86 mm^−1^
*T* = 295 K
0.26 × 0.21 × 0.16 mm

#### Data collection

Bruker SMART CCD diffractometer
Absorption correction: multi-scan (*SADABS*; Bruker, 2001 ▶) *T*_min_ = 0.801, *T*_max_ = 0.870
9547 measured reflections
3341 independent reflections
2733 reflections with *I* > 2σ(*I*)
*R*_int_ = 0.042

#### Refinement

*R*[*F* ^2^ > 2σ(*F* ^2^)] = 0.038
*wR*(*F* ^2^) = 0.082
*S* = 1.03
3341 reflections
209 parameters
1 restraint
H atoms treated by a mixture of independent and constrained refinement
Δρ_max_ = 0.55 e Å^−3^
Δρ_min_ = −0.52 e Å^−3^

### 

Data collection: *APEX2* (Bruker, 2004 ▶); cell refinement: *SAINT-Plus* (Bruker, 2001 ▶); data reduction: *SAINT-Plus*; program(s) used to solve structure: *SHELXS97* (Sheldrick, 2008 ▶); program(s) used to refine structure: *SHELXL97* (Sheldrick, 2008 ▶); molecular graphics: *SHELXTL* (Sheldrick, 2008 ▶); software used to prepare material for publication: *SHELXTL*.

## Supplementary Material

Crystal structure: contains datablocks I, global. DOI: 10.1107/S1600536810043291/hb5694sup1.cif

Structure factors: contains datablocks I. DOI: 10.1107/S1600536810043291/hb5694Isup2.hkl

Additional supplementary materials:  crystallographic information; 3D view; checkCIF report

## Figures and Tables

**Table 1:** Selected bond lengths (Å)

Cd1—O2	2.268 (2)
Cd1—O3	2.3084 (19)
Cd1—N5^i^	2.392 (2)

Symmetry code: (i) 
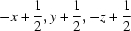
.

**Table 2:** Hydrogen-bond geometry (Å, °)

*D*—H⋯*A*	*D*—H	H⋯*A*	*D*⋯*A*	*D*—H⋯*A*
N5—H5N⋯O1^ii^	0.89 (1)	2.10 (1)	2.959 (3)	161 (3)

Symmetry code: (ii) 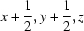.

**Fig. 1.**
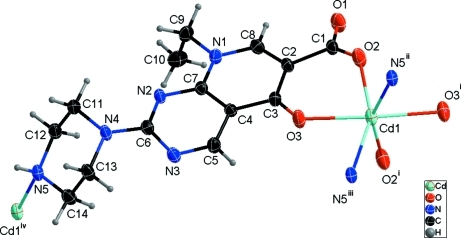
The asymmetric unit of the title compound extended to show the cadmium coordination, showing the showing 50% displacement ellipsoids.

**Fig. 2.**
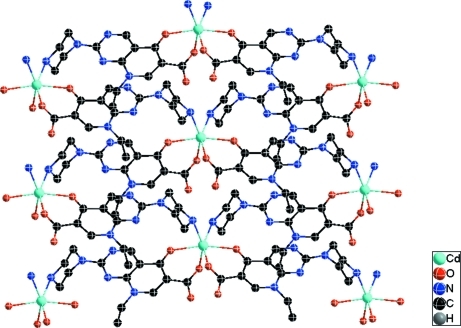
A view of part of a two-dimensional polymeric sheet in (I) showing the square-grid connectivity (H atoms omitted for clarity).

## supplementary crystallographic information

### Comment

Pipemidic acid (Hppa, C_14_H_16_N_5_O_3_, 8-Ethyl-5,8-dihydro-5-oxo-2- (1-piperazinyl)-pyrido(2,3 - d)-pyrimidine-6-carboxylic acid) is member of a class of quinolones used to treat infections (Mizuki *et al.*, 1996). Manganese(II),zinc(II), cobalt(II) and nickel(II) derivatives of ppa have been reported (Huang *et al.* 2008; Xu *et al.*2009; Qi *et al.* 2009; An & Zhu, 2010) The title cadmium(II) complex is reported here(Fig. 1).

The cadmium(II) atom is coordinated by four oxygen atoms and two N atoms from four ppa ligands (two monodentate-N and two O,*O*-bidentate) to form a square grid propagating in (Fig. 2).

### Experimental

A mixture of Cd(CH_3_COO)_2_.2H_2_O (0.13 g, 0.5 mmol), Hppa (0.15 g, 0.5 mmol), sodium hydroxide(0.04 g,1 mmol) and water (15 ml) was stirred for 30 min in air. The mixture was then transferred to a 25 ml Teflon-lined hydrothermal bomb. The bomb was kept at 433 K for 72 h under autogenous pressure. Upon cooling, colorless prisms of the title compound were obtained from the reaction mixture.

### Refinement

The carbon-bound H atoms were positioned geometrically (C—H = 0.93–0.97 Å) and were included in the refinement in the riding model approximation, with U(H) = 1.2Ueq(C). The H on Nitrogen atoms were located in a difference Fourier map, and were refined with a distance restraint of N—H = 0.86 (1) /%A and with *U*_iso_(H) = 1.2Ueq(N).

### Crystal data

**Table:**

[Cd(C_14_H_16_N_5_O_3_)_2_]	*F*(000) = 1464
*M**_r_* = 717.04	*D*_x_ = 1.739 Mg m^−^^3^
Monoclinic, *C*2/*c*	Mo *K*α radiation, λ = 0.71073 Å
Hall symbol: -C 2yc	Cell parameters from 2029 reflections
*a* = 23.565 (3) Å	θ = 2.6–25.2°
*b* = 7.4989 (10) Å	µ = 0.86 mm^−^^1^
*c* = 18.719 (3) Å	*T* = 295 K
β = 124.133 (2)°	Prism, colorless
*V* = 2738.0 (6) Å^3^	0.26 × 0.21 × 0.16 mm
*Z* = 4	

### Data collection

**Table:**

Bruker SMART CCD diffractometer	3341 independent reflections
Radiation source: fine-focus sealed tube	2733 reflections with *I* > 2σ(*I*)
graphite	*R*_int_ = 0.042
ω scans	θ_max_ = 28.1°, θ_min_ = 2.6°
Absorption correction: multi-scan (*SADABS*; Bruker, 2001)	*h* = −31→31
*T*_min_ = 0.801, *T*_max_ = 0.870	*k* = −9→9
9547 measured reflections	*l* = −24→17

### Refinement

**Table:**

Refinement on *F*^2^	Primary atom site location: structure-invariant direct methods
Least-squares matrix: full	Secondary atom site location: difference Fourier map
*R*[*F*^2^ > 2σ(*F*^2^)] = 0.038	Hydrogen site location: inferred from neighbouring sites
*wR*(*F*^2^) = 0.082	H atoms treated by a mixture of independent and constrained refinement
*S* = 1.03	*w* = 1/[σ^2^(*F*_o_^2^) + (0.0343*P*)^2^ + 1.6146*P*] where *P* = (*F*_o_^2^ + 2*F*_c_^2^)/3
3341 reflections	(Δ/σ)_max_ < 0.001
209 parameters	Δρ_max_ = 0.55 e Å^−^^3^
1 restraint	Δρ_min_ = −0.52 e Å^−^^3^

### Special details

**Table:**

Geometry. All e.s.d.'s (except the e.s.d. in the dihedral angle between two l.s. planes) are estimated using the full covariance matrix. The cell e.s.d.'s are taken into account individually in the estimation of e.s.d.'s in distances, angles and torsion angles; correlations between e.s.d.'s in cell parameters are only used when they are defined by crystal symmetry. An approximate (isotropic) treatment of cell e.s.d.'s is used for estimating e.s.d.'s involving l.s. planes.
Refinement. Refinement of *F*^2^ against ALL reflections. The weighted *R*-factor *wR* and goodness of fit *S* are based on *F*^2^, conventional *R*-factors *R* are based on *F*, with *F* set to zero for negative *F*^2^. The threshold expression of *F*^2^ > σ(*F*^2^) is used only for calculating *R*-factors(gt) *etc*. and is not relevant to the choice of reflections for refinement. *R*-factors based on *F*^2^ are statistically about twice as large as those based on *F*, and *R*- factors based on ALL data will be even larger.

### Fractional atomic coordinates and isotropic or equivalent isotropic displacement parameters (Å^2^)

**Table:**

	*x*	*y*	*z*	*U*_iso_*/*U*_eq_	
Cd1	0.0000	0.98587 (4)	0.2500	0.02310 (10)	
O1	0.08374 (10)	0.5183 (3)	0.41826 (15)	0.0332 (5)	
O2	0.04106 (10)	0.7822 (3)	0.35767 (15)	0.0344 (5)	
O3	0.11120 (10)	0.9456 (3)	0.28957 (14)	0.0317 (5)	
N1	0.26146 (11)	0.5764 (3)	0.43559 (15)	0.0219 (5)	
N2	0.33353 (11)	0.7134 (3)	0.40468 (15)	0.0216 (5)	
N3	0.29069 (12)	0.9516 (3)	0.29966 (17)	0.0262 (5)	
N4	0.40508 (11)	0.8712 (3)	0.38073 (16)	0.0235 (5)	
N5	0.49005 (11)	0.7117 (3)	0.33262 (16)	0.0220 (5)	
C1	0.08550 (13)	0.6605 (4)	0.38550 (18)	0.0222 (6)	
C2	0.14781 (13)	0.6884 (3)	0.38139 (18)	0.0201 (6)	
C3	0.15495 (13)	0.8283 (3)	0.33412 (18)	0.0204 (6)	
C4	0.21915 (13)	0.8271 (3)	0.34077 (18)	0.0192 (6)	
C5	0.23209 (14)	0.9413 (4)	0.29215 (19)	0.0246 (6)	
H5A	0.1966	1.0149	0.2518	0.030*	
C6	0.34189 (14)	0.8435 (3)	0.36193 (18)	0.0208 (6)	
C7	0.27239 (13)	0.7084 (3)	0.39339 (18)	0.0197 (6)	
C8	0.20076 (14)	0.5712 (4)	0.42765 (19)	0.0227 (6)	
H8A	0.1945	0.4798	0.4562	0.027*	
C9	0.31463 (14)	0.4398 (4)	0.48763 (19)	0.0266 (6)	
H9A	0.3068	0.3881	0.5289	0.032*	
H9B	0.3594	0.4961	0.5197	0.032*	
C10	0.3138 (2)	0.2948 (4)	0.4318 (2)	0.0468 (9)	
H10A	0.2691	0.2417	0.3985	0.070*	
H10B	0.3471	0.2055	0.4676	0.070*	
H10C	0.3248	0.3444	0.3937	0.070*	
C11	0.46394 (14)	0.7639 (4)	0.4430 (2)	0.0263 (6)	
H11A	0.4543	0.7030	0.4808	0.032*	
H11B	0.5034	0.8405	0.4781	0.032*	
C12	0.47967 (14)	0.6281 (4)	0.39627 (19)	0.0248 (6)	
H12A	0.5207	0.5626	0.4383	0.030*	
H12B	0.4421	0.5436	0.3667	0.030*	
C13	0.41713 (15)	0.9697 (4)	0.3225 (2)	0.0257 (6)	
H13A	0.4554	1.0511	0.3553	0.031*	
H13B	0.3768	1.0385	0.2815	0.031*	
C14	0.43293 (15)	0.8350 (4)	0.2749 (2)	0.0270 (7)	
H14A	0.3920	0.7653	0.2366	0.032*	
H14B	0.4443	0.8992	0.2395	0.032*	
H5N	0.5260 (10)	0.785 (3)	0.3622 (16)	0.021 (8)*	

### Atomic displacement parameters (Å^2^)

**Table:**

	*U*^11^	*U*^22^	*U*^33^	*U*^12^	*U*^13^	*U*^23^
Cd1	0.01777 (14)	0.02430 (17)	0.02992 (18)	0.000	0.01502 (13)	0.000
O1	0.0289 (11)	0.0292 (12)	0.0461 (14)	−0.0023 (9)	0.0239 (11)	0.0077 (10)
O2	0.0284 (12)	0.0353 (12)	0.0484 (15)	0.0102 (9)	0.0270 (11)	0.0151 (11)
O3	0.0223 (11)	0.0349 (12)	0.0430 (14)	0.0074 (9)	0.0213 (10)	0.0144 (10)
N1	0.0186 (11)	0.0210 (12)	0.0262 (14)	0.0015 (9)	0.0126 (11)	0.0024 (10)
N2	0.0175 (11)	0.0250 (12)	0.0251 (14)	−0.0003 (9)	0.0137 (11)	0.0014 (10)
N3	0.0244 (12)	0.0286 (13)	0.0311 (14)	0.0043 (10)	0.0189 (12)	0.0075 (11)
N4	0.0194 (12)	0.0262 (13)	0.0304 (14)	0.0025 (9)	0.0175 (11)	0.0051 (11)
N5	0.0171 (12)	0.0224 (12)	0.0289 (14)	−0.0009 (9)	0.0144 (11)	−0.0003 (10)
C1	0.0170 (13)	0.0259 (15)	0.0244 (16)	−0.0054 (11)	0.0121 (13)	−0.0030 (12)
C2	0.0199 (13)	0.0201 (13)	0.0233 (15)	−0.0036 (10)	0.0140 (12)	−0.0015 (11)
C3	0.0175 (13)	0.0207 (14)	0.0230 (15)	−0.0011 (11)	0.0113 (12)	−0.0032 (11)
C4	0.0189 (13)	0.0195 (13)	0.0207 (15)	−0.0002 (10)	0.0121 (12)	0.0010 (11)
C5	0.0213 (14)	0.0248 (14)	0.0285 (17)	0.0028 (11)	0.0144 (13)	0.0036 (12)
C6	0.0212 (14)	0.0188 (13)	0.0257 (16)	0.0005 (11)	0.0152 (13)	−0.0016 (11)
C7	0.0185 (13)	0.0200 (14)	0.0207 (15)	−0.0021 (10)	0.0111 (12)	−0.0020 (11)
C8	0.0245 (14)	0.0205 (14)	0.0267 (16)	−0.0036 (11)	0.0166 (13)	−0.0010 (12)
C9	0.0225 (14)	0.0253 (15)	0.0282 (17)	0.0057 (11)	0.0119 (14)	0.0093 (12)
C10	0.059 (2)	0.0329 (19)	0.044 (2)	0.0184 (17)	0.026 (2)	0.0076 (16)
C11	0.0204 (14)	0.0343 (16)	0.0253 (16)	0.0028 (12)	0.0135 (13)	0.0041 (13)
C12	0.0209 (14)	0.0265 (15)	0.0285 (17)	0.0016 (11)	0.0148 (13)	0.0045 (13)
C13	0.0255 (14)	0.0221 (14)	0.0377 (18)	0.0026 (11)	0.0227 (14)	0.0076 (13)
C14	0.0287 (15)	0.0270 (15)	0.0326 (18)	0.0045 (12)	0.0216 (15)	0.0075 (13)

### Geometric parameters (Å, °)

**Table:**

Cd1—O2^i^	2.268 (2)	C2—C8	1.366 (4)
Cd1—O2	2.268 (2)	C2—C3	1.442 (4)
Cd1—O3^i^	2.3084 (19)	C3—C4	1.447 (3)
Cd1—O3	2.3084 (19)	C4—C7	1.396 (4)
Cd1—N5^ii^	2.392 (2)	C4—C5	1.402 (4)
Cd1—N5^iii^	2.392 (2)	C5—H5A	0.9300
O1—C1	1.243 (3)	C8—H8A	0.9300
O2—C1	1.260 (3)	C9—C10	1.501 (4)
O3—C3	1.252 (3)	C9—H9A	0.9700
N1—C8	1.353 (3)	C9—H9B	0.9700
N1—C7	1.378 (3)	C10—H10A	0.9600
N1—C9	1.483 (3)	C10—H10B	0.9600
N2—C7	1.334 (3)	C10—H10C	0.9600
N2—C6	1.344 (3)	C11—C12	1.518 (4)
N3—C5	1.309 (3)	C11—H11A	0.9700
N3—C6	1.376 (3)	C11—H11B	0.9700
N4—C6	1.340 (3)	C12—H12A	0.9700
N4—C11	1.454 (4)	C12—H12B	0.9700
N4—C13	1.470 (3)	C13—C14	1.524 (4)
N5—C12	1.483 (3)	C13—H13A	0.9700
N5—C14	1.484 (3)	C13—H13B	0.9700
N5—Cd1^iv^	2.392 (2)	C14—H14A	0.9700
N5—H5N	0.893 (10)	C14—H14B	0.9700
C1—C2	1.527 (3)		
			
O2^i^—Cd1—O2	95.31 (12)	N4—C6—N2	117.9 (2)
O2^i^—Cd1—O3^i^	77.61 (7)	N4—C6—N3	116.6 (2)
O2—Cd1—O3^i^	92.21 (7)	N2—C6—N3	125.4 (2)
O2^i^—Cd1—O3	92.21 (7)	N2—C7—N1	117.5 (2)
O2—Cd1—O3	77.61 (7)	N2—C7—C4	123.8 (2)
O3^i^—Cd1—O3	164.98 (10)	N1—C7—C4	118.6 (2)
O2^i^—Cd1—N5^ii^	92.86 (8)	N1—C8—C2	125.8 (3)
O2—Cd1—N5^ii^	154.65 (8)	N1—C8—H8A	117.1
O3^i^—Cd1—N5^ii^	112.99 (8)	C2—C8—H8A	117.1
O3—Cd1—N5^ii^	78.14 (7)	N1—C9—C10	111.6 (3)
O2^i^—Cd1—N5^iii^	154.65 (8)	N1—C9—H9A	109.3
O2—Cd1—N5^iii^	92.86 (8)	C10—C9—H9A	109.3
O3^i^—Cd1—N5^iii^	78.14 (7)	N1—C9—H9B	109.3
O3—Cd1—N5^iii^	112.99 (8)	C10—C9—H9B	109.3
N5^ii^—Cd1—N5^iii^	89.87 (11)	H9A—C9—H9B	108.0
C1—O2—Cd1	134.12 (18)	C9—C10—H10A	109.5
C3—O3—Cd1	131.89 (17)	C9—C10—H10B	109.5
C8—N1—C7	119.0 (2)	H10A—C10—H10B	109.5
C8—N1—C9	120.2 (2)	C9—C10—H10C	109.5
C7—N1—C9	120.8 (2)	H10A—C10—H10C	109.5
C7—N2—C6	115.6 (2)	H10B—C10—H10C	109.5
C5—N3—C6	115.3 (2)	N4—C11—C12	110.0 (2)
C6—N4—C11	123.0 (2)	N4—C11—H11A	109.7
C6—N4—C13	122.1 (2)	C12—C11—H11A	109.7
C11—N4—C13	112.2 (2)	N4—C11—H11B	109.7
C12—N5—C14	110.9 (2)	C12—C11—H11B	109.7
C12—N5—Cd1^iv^	109.91 (16)	H11A—C11—H11B	108.2
C14—N5—Cd1^iv^	110.40 (17)	N5—C12—C11	112.5 (2)
C12—N5—H5N	106.7 (19)	N5—C12—H12A	109.1
C14—N5—H5N	103.1 (19)	C11—C12—H12A	109.1
Cd1^iv^—N5—H5N	115.7 (18)	N5—C12—H12B	109.1
O1—C1—O2	125.1 (2)	C11—C12—H12B	109.1
O1—C1—C2	116.2 (2)	H12A—C12—H12B	107.8
O2—C1—C2	118.7 (2)	N4—C13—C14	108.2 (2)
C8—C2—C3	118.5 (2)	N4—C13—H13A	110.1
C8—C2—C1	116.2 (2)	C14—C13—H13A	110.1
C3—C2—C1	125.2 (2)	N4—C13—H13B	110.1
O3—C3—C2	125.7 (2)	C14—C13—H13B	110.1
O3—C3—C4	119.5 (2)	H13A—C13—H13B	108.4
C2—C3—C4	114.8 (2)	N5—C14—C13	114.0 (2)
C7—C4—C5	114.2 (2)	N5—C14—H14A	108.7
C7—C4—C3	123.2 (2)	C13—C14—H14A	108.7
C5—C4—C3	122.6 (2)	N5—C14—H14B	108.7
N3—C5—C4	124.5 (3)	C13—C14—H14B	108.7
N3—C5—H5A	117.7	H14A—C14—H14B	107.6
C4—C5—H5A	117.7		
			
O2^i^—Cd1—O2—C1	61.3 (3)	C13—N4—C6—N3	−18.1 (4)
O3^i^—Cd1—O2—C1	139.0 (3)	C7—N2—C6—N4	170.3 (2)
O3—Cd1—O2—C1	−29.8 (3)	C7—N2—C6—N3	−9.9 (4)
N5^ii^—Cd1—O2—C1	−47.0 (4)	C5—N3—C6—N4	−169.7 (3)
N5^iii^—Cd1—O2—C1	−142.7 (3)	C5—N3—C6—N2	10.6 (4)
O2^i^—Cd1—O3—C3	−80.2 (3)	C6—N2—C7—N1	179.6 (2)
O2—Cd1—O3—C3	14.7 (3)	C6—N2—C7—C4	0.4 (4)
O3^i^—Cd1—O3—C3	−33.5 (3)	C8—N1—C7—N2	177.9 (2)
N5^ii^—Cd1—O3—C3	−172.7 (3)	C9—N1—C7—N2	−3.2 (4)
N5^iii^—Cd1—O3—C3	102.6 (3)	C8—N1—C7—C4	−2.9 (4)
Cd1—O2—C1—O1	−148.5 (2)	C9—N1—C7—C4	176.0 (2)
Cd1—O2—C1—C2	33.1 (4)	C5—C4—C7—N2	7.0 (4)
O1—C1—C2—C8	−11.5 (4)	C3—C4—C7—N2	−175.3 (3)
O2—C1—C2—C8	167.1 (3)	C5—C4—C7—N1	−172.2 (2)
O1—C1—C2—C3	169.4 (3)	C3—C4—C7—N1	5.5 (4)
O2—C1—C2—C3	−12.0 (4)	C7—N1—C8—C2	−0.8 (4)
Cd1—O3—C3—C2	−6.4 (4)	C9—N1—C8—C2	−179.7 (3)
Cd1—O3—C3—C4	173.52 (18)	C3—C2—C8—N1	2.0 (4)
C8—C2—C3—O3	−179.5 (3)	C1—C2—C8—N1	−177.2 (3)
C1—C2—C3—O3	−0.4 (5)	C8—N1—C9—C10	98.4 (3)
C8—C2—C3—C4	0.6 (4)	C7—N1—C9—C10	−80.4 (3)
C1—C2—C3—C4	179.6 (2)	C6—N4—C11—C12	101.2 (3)
O3—C3—C4—C7	175.7 (3)	C13—N4—C11—C12	−60.4 (3)
C2—C3—C4—C7	−4.3 (4)	C14—N5—C12—C11	−50.3 (3)
O3—C3—C4—C5	−6.7 (4)	Cd1^iv^—N5—C12—C11	−172.71 (18)
C2—C3—C4—C5	173.2 (3)	N4—C11—C12—N5	55.3 (3)
C6—N3—C5—C4	−1.7 (4)	C6—N4—C13—C14	−102.7 (3)
C7—C4—C5—N3	−6.3 (4)	C11—N4—C13—C14	59.0 (3)
C3—C4—C5—N3	176.0 (3)	C12—N5—C14—C13	50.8 (3)
C11—N4—C6—N2	1.9 (4)	Cd1^iv^—N5—C14—C13	172.90 (17)
C13—N4—C6—N2	161.7 (2)	N4—C13—C14—N5	−54.3 (3)
C11—N4—C6—N3	−177.9 (3)		

Symmetry codes: (i) −*x*, *y*, −*z*+1/2; (ii) −*x*+1/2, *y*+1/2, −*z*+1/2; (iii) *x*−1/2, *y*+1/2, *z*; (iv) *x*+1/2, *y*−1/2, *z*.

### Hydrogen-bond geometry (Å, °)

**Table:**

*D*—H···*A*	*D*—H	H···*A*	*D*···*A*	*D*—H···*A*
N5—H5N···O1^v^	0.89 (1)	2.10 (1)	2.959 (3)	161 (3)

Symmetry codes: (v) *x*+1/2, *y*+1/2, *z*.
